# Chemical and Biological Studies of *Cannabis sativa* Roots

**DOI:** 10.1159/000495582

**Published:** 2018-12-04

**Authors:** Mostafa A. Elhendawy, Amira S. Wanas, Mohamed M. Radwan, Nabil A. Azzaz, ElShahat S. Toson, Mahmoud A. ElSohly

**Affiliations:** a National Center for Natural Products Research, School of Pharmacy, University of Mississippi, University, MS, USA;; b Department of Chemistry, Faculty of Agriculture, Damietta University, Damietta, Egypt;; c Department of Pharmacognosy, Faculty of Pharmacy, Minia University, Minia, Egypt;; d Department of Chemistry, Faculty of Science, Damietta University, Damietta, Egypt;; e Department of Pharmaceutics and Drug Delivery, School of Pharmacy, University of Mississippi, University, MS, USA

**Keywords:** Cannabis sativa, Secondary metabolites, High-performance liquid chromatography, Quantitative analysis, Cannabis roots

## Abstract

The chemical study of *Cannabis sativa* roots led to the isolation and identification of 10 compounds. Their chemical structures were unambiguously established on the basis of 1D and 2D NMR spectroscopy and mass spectrometry as friedelan-3-one (1), epifriedelanol (2), β-sitosterol (3), ergost-5-en-3-ol (4), methyl hexadecanoate (5), pentadecanoic acid (6), 10E-hexadecenoic acid (7), 4-hydroxy-3-methoxybenzaldehyde (8), β-sitosterol-β-D-glucoside (9) and *p*-coumaroyltyramine (10). Compounds 5–9 were reported for the first time from *C. sativa* roots. All the isolated compounds were tested for their antimicrobial activity. Compound 4 showed modest activity against *Cryptococcus neoformans* with an IC_50_ value of 13.7 μg/mL, while compound 10 displayed potent activity against *Escherichia coli* with an IC_50_ value of 0.8 μg/mL. A high-performance liquid chromatography method was developed and validated for the detection and quantification of *p*-coumaroyltyramine (10) in the extracts of different varieties of *C. sativa* roots.

## Introduction

*Cannabis sativa* L. is one of the most widely used plants for both recreational and medicinal purposes. To date, a total of 567 natural constituents covering several chemical classes have been identified from *C. sativa* [1, 2]. The most important classes are the cannabinoids, terpenoids, nitrogenous compounds, noncannabinoid phenols, flavonoids, and steroids [3]. The principal use of cannabis in medicine is for easing pain and in ameliorating nervous system disorders. It is reported to be useful in the treatment of gout, neuralgia, rheumatism, insanity, and insomnia among others, with actions almost entirely on the higher nerve centers [4].

The first reference to cannabis consumption dates as far back as 2,700 BC in China, with *Shennong pên Ts’aoching*, one of the oldest Chinese medicine books, mentioning the use of cannabis roots as a remedy to sooth pain. Throughout history, cannabis roots were documented in ancient Greek medicine, and a medical article in how Indians boiled them together with other leaves to make poultices for the treatment of inflamed skin surfaces and skin rash [3, 5]. There are numerous reports on the traditional use of cannabis root for the treatment of fever, inflammation, gout, arthritis, and joint pain, as well as skin burns and hard tumors [3]. Also, they were used to treat postpartum hemorrhage, difficult child labor, sexually transmitted disease, and gastrointestinal activity and infection [6]. Despite a long history of therapeutic use, the roots of cannabis plants have been ignored in modern medical research and practice. Cannabis roots have been reported to have many different compounds, including triterpenoids, monoterpenes, alkaloids, sterols, amides, and choline [3].

On continuation of our search for bioactive compounds from *C. sativa* [7, 8], this article describes the isolation and structural elucidation as well as the antimicrobial activity of 10 compounds from *C. sativa* roots. A high-performance liquid chromatography (HPLC) method was also developed and validated for the quantification of *p*-coumaroyltyramine (10) in extracts of different varieties of *C. sativa* roots which could be used as a possible marker compound to distinguish between roots of different varieties of *C. sativa*.

## Materials and Methods

### General Experimental Procedures

1D and 2D NMR spectra were recorded using the residual solvent signal as an internal standard on Bruker BioSpin Gm bH 400 and 500 NMR spectrometers (Bruker, Rheinstetten, Germany). Thin-layer chromatography (TLC) was carried out on aluminum-packed plates precoated with silica gel F_254_ (Silicycle, Quebec, QC, Canada). Visualization was accomplished by spraying with a vanillin sulfuric acid spray reagent followed by heating.

### Plant Material

*C. sativa* plants were grown at the University of Mississippi, USA and identified by Dr. Suman Chandra, senior scientist, NCNPR, School of Pharmacy, University of Mississippi. The fresh roots were washed with tap water followed by distilled water and dried under shade. The dried roots were powdered using a coffee grinder.

### Extraction and Isolation

The dried ground roots of a high cannabidiol (CBD) variety of cannabis (2.0 kg) were sequentially extracted with hexanes (2 × 16 L), dichloromethane (C_2_Cl_2_) (20 L), ethyl acetate (EtOAc) (20 L), and methanol (MeOH) (20 L) at room temperature. The extracts were evaporated under reduced pressure at 40 ° C to afford hexanes (3.8 g), CH_2_Cl_2_ (3.5 g), EtOAc (1.4 g) and MeOH (0.5 g) extracts. The hexanes extract (3.8 g) was subjected to silica gel column chromatography (75 × 2.5 cm i.d.), eluted with EtOAc/n-hexane (0:100, 10:90, 20:80, 30:70, 40:60, 50: 50, 75:25, 100:0 v/v [1.0 L of each mixture]), yielding 10 fractions (H_1_-H_10_). Fraction Hi (197.8 mg) was applied to a C18 solid-phase extraction column eluted with MeOH/H_2_O (75:25), to afford compound 1 (127 mg) and compound 2 (46.3 mg). Fraction H_5_ (717.9 mg) was fractionated over a silica gel column (45 × 2 cm, 50 g) and eluted with EtOAc/n-hexane (0:100–20:80, 5% stepwise) to afford compound 3 and 4 subfractions (A–D). Subfraction A (54.4 mg) was purified on a 5.0 g solid-phase extraction amino column eluted with CH_2_Cl_2_ (50 mL), 5% isopropanol/CH_2_Cl_2_ (50 mL), 2% acetic acid/diethyl ether (50 mL) and MeOH (50 mL) to yield compound 4 (21 mg). The CH_2_Cl_2_ and EtOAc extracts were mixed together (4.9 g) based on TLC analysis; subjected to silica gel column chromatography (100 × 3.5 cm, 150 g), using EtOAc/CH_2_Cl_2_ as eluant (0:100–100: 0, 5% stepwise), to yield 10 fractions (DE_1_-DE_10_). Fraction DE_1_ (104.2 mg) was chromatographed over a silica gel column (50 6× 1 cm, 19 g) and eluted with chloroform (CHCl_3_)/n-hexane mixture (0:100, 30:70, 50:50, 70:30, 100:0 [80 mL of each mixture]), to yield compound 5 (20 mg). Fraction DE2 (237.9 mg) was chromatographed on a silica gel column using 100% dichloromethane as a mobile phase to afford compounds 6 (17.7 mg), 7 (63.7 mg) and 8 (15.2 mg). Fraction DE_9_ (540.5 mg) was applied to a C_18_ RP silica column (30 × 2 cm, 50 g) eluting with MeOH/H_2_O (75:25, 80: 20, 90:10, 100:0), to afford compounds 9 (25 mg) and 10 (8.2 mg).

### In vitro Antimicrobial Assay

All organisms used for the biological evaluation were obtained from the American Type Culture Collection (Manassas, VA, USA). These include the fungi *Candida albicans* ATCC 90028, *Cryptococcus neoformans* ATCC 90113, and *Aspergillus fumigatus* ATCC 90906 and the bacteria methicillin-resistant *Staphylococcus aureus* ATCC 43300 (MRS), *Escherichia coli* ATCC 35218, *Pseudomonas aeruginosa* ATCC 27853, *Klebsiella pneumoniae* ATCC 25955, and vancomycin-resistant enterococcus. Susceptibility testing was performed using a modified version of the CLSI (formerly NCCLS) methods as previously described [9, 10].

### HPLC Analysis of p-Coumaroyltyramine (10)

#### Reagents and Materials

**Fig. 1.**
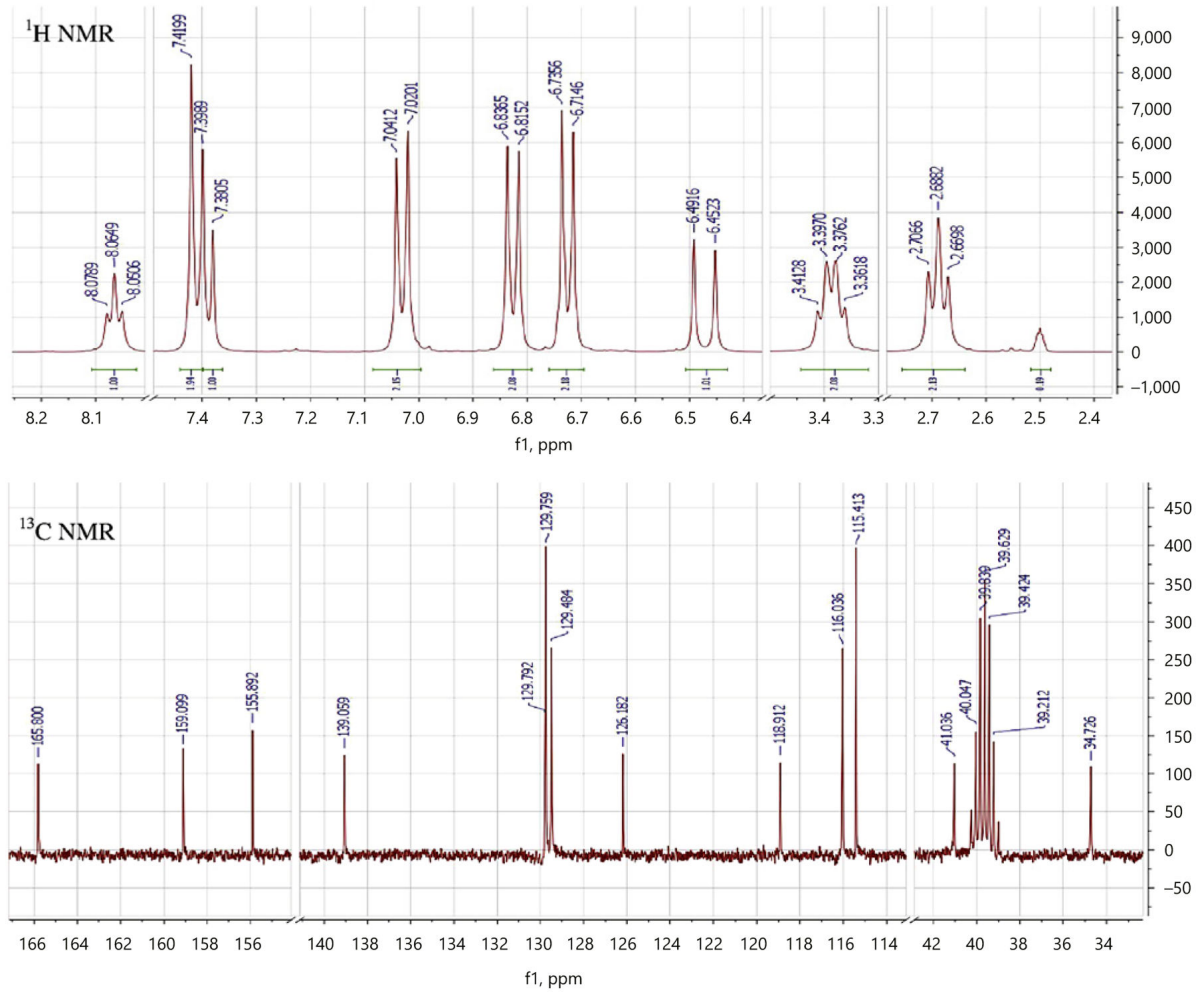
^1^H (400 MHz) and ^13^C NMR (100 MHz) spectra of compound 10.

Acetonitrile (CH_3_CN), MeOH, tetrahydrofuran, and H_2_O are of HPLC grade (Fisher Scientific, Fairlawn, NJ, USA). Compound 10 was isolated from the roots of *C. sativa*. The chemical structure of compound 10 was identified by ^1^H NMR, ^13^C NMR, heteronuclear multiple-quantum correlation, heteronuclear multiple-bond correlation, and electrospray ionization mass spectrometry. The ^1^H and ^13^C NMR data of compound 10 are shown in Table 1 and Figure 1. The purity of compound 10 was determined on the basis of UV, NMR, MS and HPLC to be >98%.

#### Apparatus and Chromatographic Conditions

A Waters 9526 HPLC system equipped with a quaternary solvent delivery system, an autosampler and a DAD detector were used. Separation was achieved on a Phenomenex luna C18 column (250 × 4.6. mm i.d., 5 μm particle size). The mobile phase consisted of (a) CH_3_CN:MeOl·l (1:1) and (b) H_2_O. The gradient elution (% a in b) was as follows: 0–8 min, linear gradient from 30 to 45%; 8–10 min, linear gradient to 50%; 10–15 min, linear gradient to 60%, which was held for 5 min then returned to 30%. Each run was followed by equilibration time of 5 min. The flow rate was 1.0 mL/min and the total run time was 20 min. The column temperature was set at 30° C. DAD spectra were monitored from 210 to 480 nm and the detection wavelength was set to λ_max_ 290 nm. The injection volume was 10 μL.

#### Preparation of Standard Solutions and Calibration Curve

A 1.0 mg/mL stock solution of compound 10 in MeOH was prepared and diluted to an appropriate concentration range to establish the calibration curve. The calibration curve was constructed at 1, 5, 10, 25, 50, 75 and 100 μg/mL each in triplicate.

#### Preparation of Sample Solutions

Three different varieties of *C. sativa* roots (high CBD, intermediate, high tetrahydrocannabinol [THC] variety) were separately ground. Each sample (1.0 g) was accurately weighed and extracted with 10 mL tetrahydrofuran by sonication for 30 min and 5.0 mL of the extract was dried with N_2_. The final volume was adjusted to 1.0 mL with MeOH, filtered through a 0.45-μm filter and the filtrate injected into the HPLC system.

#### Method Validation

The method was validated for linearity, precision (interday, intraday and intermediate precision), accuracy, stability, specificity, and selectivity following the International Conference on Harmonization (ICH) guideline [11].

## Results and Discussion

### Identification of the Isolated Compounds

**Fig. 2.**
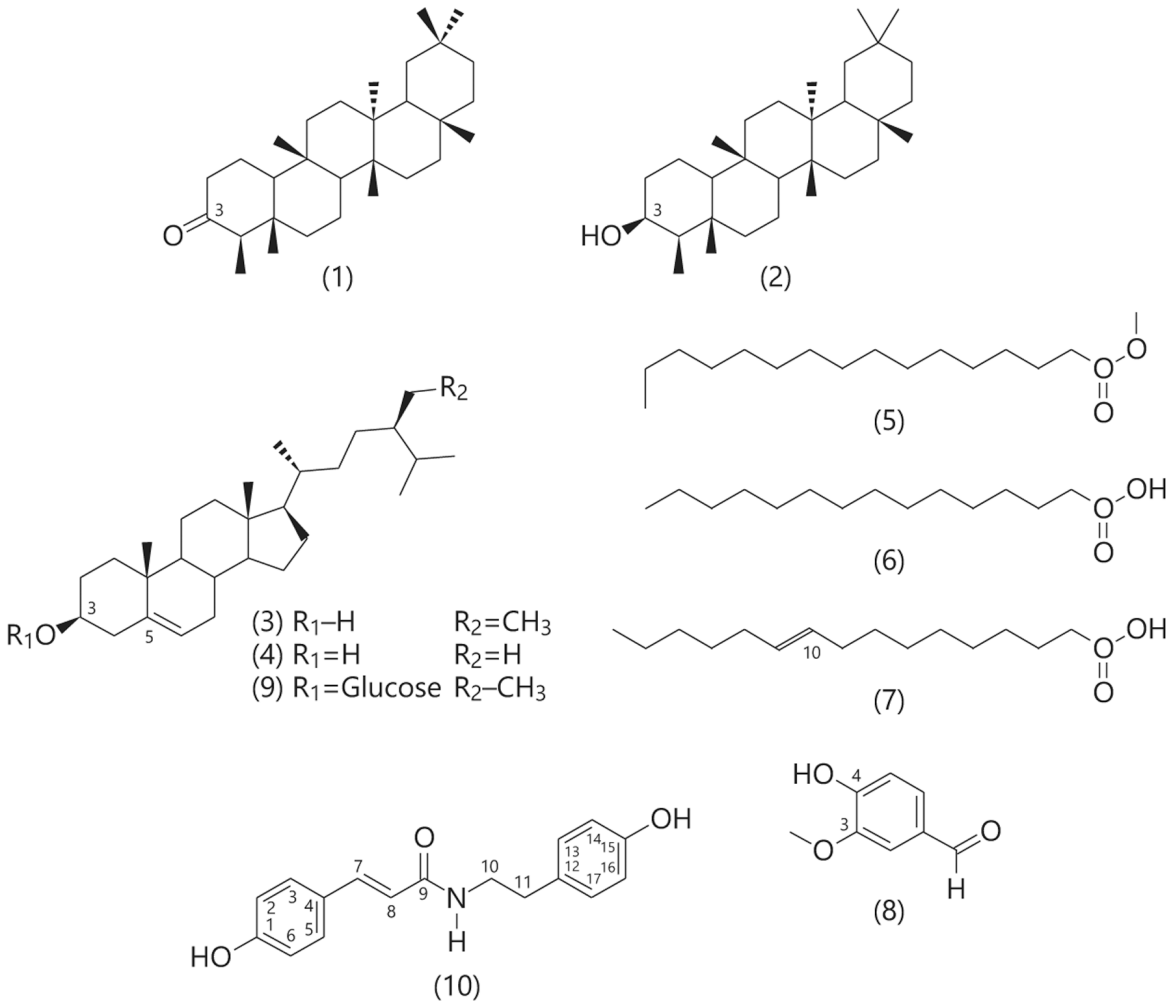
Structure of compounds 1–10 isolated from *C. sativa* roots.

Compounds 1–10 (Fig. 2) were identified by comparing their spectroscopic characteristics with those previously reported in the literature as friedelan-3-one (1) [12], epifriedelanol (2) [12], β-sitosterol (3) [13], ergost-5-en-3-ol (4) [14], methyl hexadecanoate (5) [15], pentadecanoic acid (6) [16], 10E-hexadecenoic acid (7) [16], 4-hydroxy-3-methoxybenzaldehyde (8) [17], and β-sitosterol-β-D-glucoside (9) [18]. Compound 10 was isolated as white amorphous powder. Its positive reaction using the ferric chloride test showed the phenolic nature of this compound. The molecular formula of 10 was deduced as C_1 7_H_1 7_O_3_N from LRESIMS analysis which showed a protonated molecular ion [M+H]^+^ at *m/z* 284 in the positive ionized mode. The ^13^C NMR spectrum of 10 (Table 1; Fig. 1) showed 13 carbon signals which were sorted by distortionless enhancement by polarization transfer and heteronuclear multiple-quantum correlation techniques as two methylenes (δ_C_ 34.7/δ_H_ 2.69; δ_C_ 41.0/δ_H_ 3.38) and six methynes (δ_C_ 116/δ_H_ 6.81; δ_C_ 115.4/δ_H_ 6.71; δ_C_ 118.9/δ_H_ 6.45/; δ_C_ 129.5/δ_H_ 7.02; δ_C_ 129.7/δ_H_ 7.39; δC 139/δH 7.38). The five remaining carbon signals were attributed to quaternary carbons among which one amide carbonyl at δ_C_ 165.8 and two oxygenated aromatic carbon at δ_C_ 159.1 and δ_C_ 155.9. The ^1^H NMR spectrum of 10 exhibited 8 proton signals which were analyzed using correlation spectroscopy spectrum that showed two AA’BB’ system attributed to two *para* substituted aromatic moieties, respectively, at δ_H_ 7.02 and δ_H_ 6.71 (*J* = 8.4 Hz); δ_H_ 7.39 and δ_H_ 6.81 (*J* = 8.4 Hz); one pair of doublet of *trans* substituted ethylene moiety protons at δ_H_ 7.38 and δ_H_ 6.45 (*J* = 16.0 Hz) and one multiple and one triplet of two protons each attributed to 1,2-disubstituted ethane moiety at δ_H_ 3.38 and δ_H_ 2.69 (*J* = 7.4 Hz). The hetero-nuclear multiple-bond correlation between the ethylene proton (δ_H_ 7.38, H-7) with both the amide carbonyl (δ_C_ 165.8, C-9) and the aromatic carbon (δ_C_ 129.5, C-3 and C5) confirms the presence of the *trans*-coumaroyl moiety, while the heteronuclear multiple-bond correlation between the ethyl proton (δ_H_ 2.69, H-10) with both the amide carbonyl (δC 165.8, C-9) and the quaternary aromatic carbon at δ_C_ 129.8 (C-12) supports the presence of the tyramine moiety. Based on the above data and comparing with literature [19], the chemical structure of compound 10 was elucidated to be N-*p*-*trans*-coumaroyltyramine.

### Antimicrobial Activity

The antimicrobial activities of all isolated compounds were determined against methicillin-resistant *S. aureus* (MRSa), *E. coli*, *P. aeruginosa*, and *Mycobacterium intra-cellulare*, as well as against pathogenic fungi including *C. albicans, A. fumigatus*, and *C. neoformans*. Compounds 4 and 10 showed antimicrobial activity. Compound 4 was active against *C. neoformans* with an IC_50_ value of 13.7 μg/mL, while compound 10 was active against *E. coli* with an IC50 value of 0.8 μg/mL (Table 2).

### HPLC Analysis

Plant constituents vary considerably based on several factors such as temperature, light, drying, packing, and storage, which may impair not only the quality of phyto-therapeutic agents but also their therapeutic value [20]. Thus, standardization of raw materials and herbal preparations needs to be permanently carried out in term of quality specification, stability profiles and chemical analysis of analytes of interest using sensitive validated analytical methods [21]. HPLC is a unique, versatile, universal and well-recognized tool for qualitative and quantita tive evaluation of herbal products against their respective bioactive molecules in terms of quality and batch-to-batch reproducibility [22]. Thus, in this study contribution, we have developed a simple, economic and rapid chromatographic method using RP-HPLC for the estimation of *p*-coumaroyltyramine (10) in different varieties of *C. sativa* roots.

### Method Validation

**Fig. 3.**
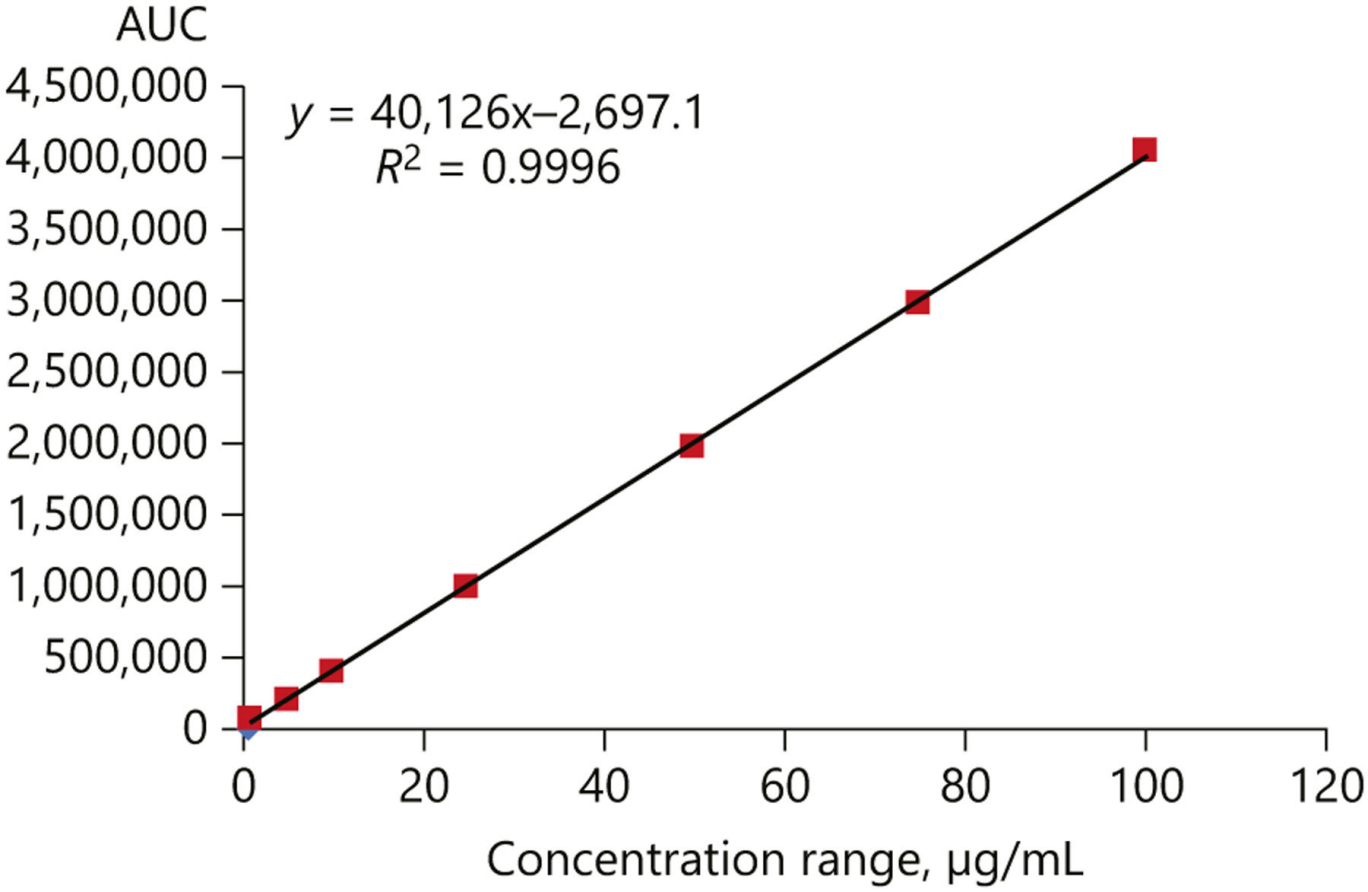
Calibration curve of *p*-coumaroyltyramine (compound 10).

Compound 10 was detected and quantified by HPLC, using a gradient mobile phase consisting ofCH_3_CN:MeOH (1:1) and H_2_O. Compound 10 showed a sharp peak at 8.81 ± 0.015 min under the optimized chromatographic conditions at λ_max_ 290 nm. Representative chromatograms are depicted in Figure 3. The separation of the marker compound (10) in a short time enabled rapid analysis of the samples. The calibration curve showed good linearity relationship in the specified concentration range (1–100 μg/mL) with a correlation coefficient (*r*^2^) of 0.9996 (Fig. 3; Table 3). The limits of detection and quantification were found to be 0.025 μg/mL and 0.1 μg/mL, respectively, thus suggesting a high sensitivity of the method which can be successfully exploited for quantifying even low sample concentrations of compound 10 (Table 3). The relative standard deviation for system suitability in terms of R_t_ and area were found to be less than 6% indicating the stability of the chromatographic method. The percent relative standard deviation of inter- and intraday analysis of standard and extract were also found to be less than 7 with a high repeatability of both R_t_ and response (Table 3). Mean recovery for the quality control samples of *p*-coumaroyltyramine (10) was found to be >98% (Table 4). Because of the almost quantitative recovery of compound 10 and the consistency of the analysis, the external standard method was adopted for quantification.

### Method Application

**Fig. 4.**
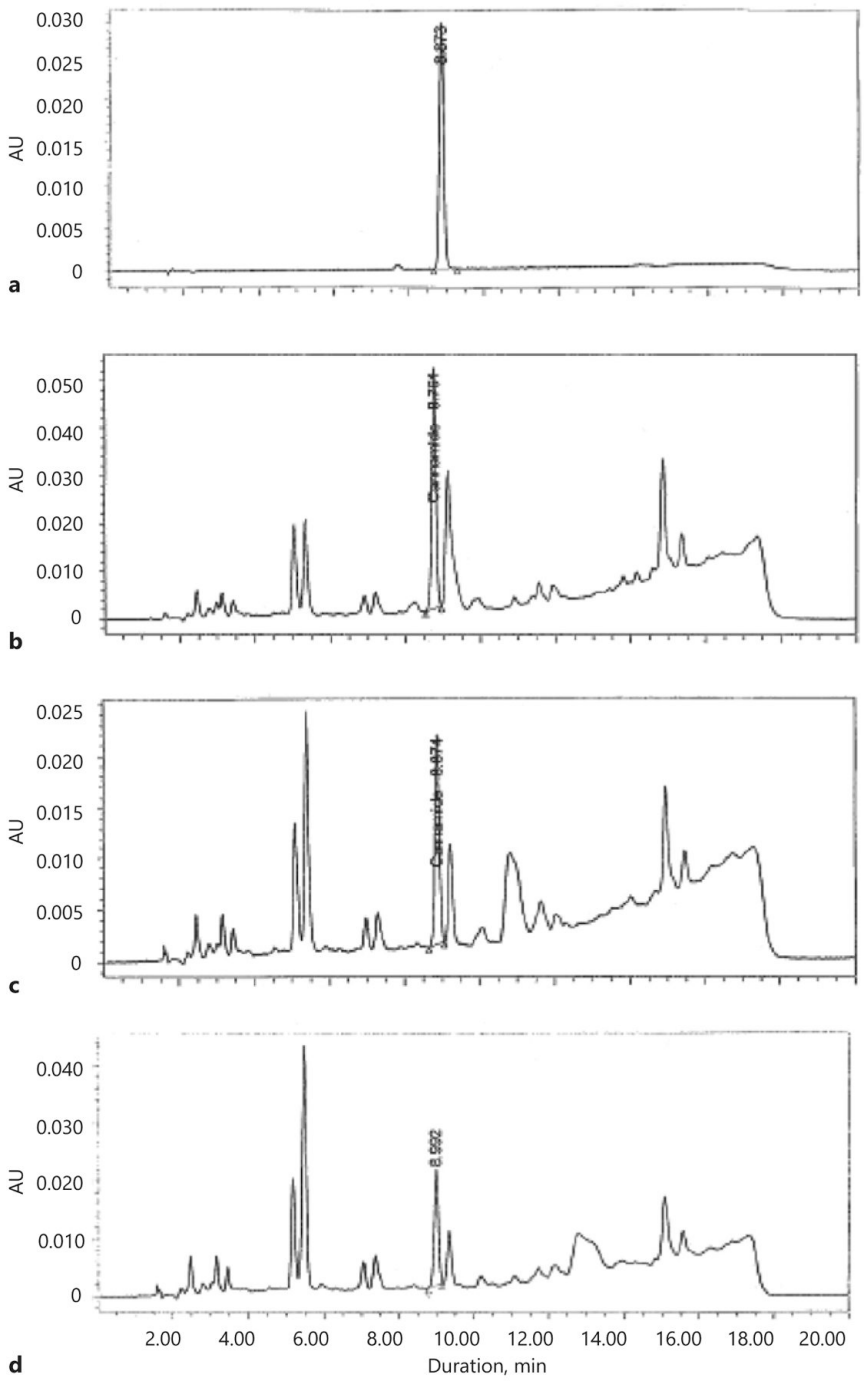
Typical HPLC profiles of a 5 μg/mL standard *p*-coumaroyltyramine (10) (**a**), and *C. sativa* extracts (**b-d**) of a high CBD variety (**b**), intermediate variety (**c**), and high THC variety (**d**).

The validated method was employed for the quantitation of *p*-coumaroyltyramine (10) from different varieties of *C. sativa* roots, namely high CBD, intermediate and high THC varieties. The HPLC profiles of the cannabis extracts samples showed a sharp peak for compound 10 at R_t_ 8.81 (± 0.015) min comparable to the standard. Figure 4 demonstrates a clear baseline separation of compound 10 in the three varieties of cannabis from adjacent peaks. The content of compound 10 in the high CBD variety was 19.78 ± 0.728 μg/g, while in the intermediate and high THC varieties it was 8.00 ± 0.348 and 7.65 ± 0.359 μg/g, respectively. The representative chromatograms and values are shown in Figure 4 and Table 5, respectively.

## Conclusion

Ten compounds have been isolated and identified from a high CBD variety of cannabis roots, of which compounds 4 and 10 showed promising antimicrobial activities. A validated HPLC method for the quantitation of compound 10 in three varieties of *C. sativa* was developed. The method was fast, simple and accurate and could be used for routine analysis of this marker compound in cannabis roots. Whether this compound can be used as a marker to discriminate between CBD variety roots and the other two varieties need to be further investigated.

## Figures and Tables

**Table 1.** ^l^H (400 MHz) and ^13^C NMR (100 MHz) spectroscopic data of *p*-coumaroyltyramine (compound 10) (DMSO)^a^

Carbon	Compound 10	
	δ_C_	δ_H_, mult. (*J* in Hz)
1	159.1	
2, 6	115.4	6.71, d (8.4)
3, 5	129.5	7.02, d (8.4)
4	126.2	
7	139.1	7.38, d (16.0)
8	118.9	6.45, d (16.0)
9	165.8	
10	34.7	2.69, d (7.4)
11	41.0	3.38, m
12	129.8	
13, 17	129.7	7.39, d (8.4)
14, 16	116.0	6.81, d (8.4)
15	155.9	
NH		8.06, t (5.7)

a Assignment conformed by distortionless enhancement by polarization transfer 135, heteronuclear multiple-quantum correlation, COSY and heteronuclear multiple-bond correlation NMR experiment.

**Table 2.** In vitro antimicrobial activities of compound 4 and 10 (IC50 in μg/mL)

Compound	Antifungal *C. neoformans*	Antibacterial *E. coli*
4	13.67	na
10	na	0.8
Ciprofloxacin	na	0.01
Amphotericin B	1.29	na

IC_50_, the concentration that causes 50% inhibition of growth; na, not active.

**Table 3.** Results of validation of *p*-coumaroyltyramine (compound 10) using HPLC in terms of linearity, sensitivity, and precision

	Parameters
*Linearity (n = 3)*	
Concentration range, μg/mL	0.25–100
Regression equation	Y = 40126X-2697.1
Correlation coefficient (*r*^2^)	0.9996
*Sensitivity*	
LOD, μg/mL	0.25
LOQ, μg/mL	0.75
*Precision (n = 6)*	

	Concentration, μg/mL		Area		R_t_, min	
	mean ± SD	% RSD	mean ± SD	% RSD	mean ± SD	% RSD
Intraday						
1st day	22.03±1.25	5.69	439,467±25,147.32	5.72	8.80±0.037	0.427
2nd day	23.40±0.24	1.07	464,383.66±5,000.918	1.08	8.76±0.015	0.176
3rd day	20.52±0.99	4.87	409,044.66±20,060.68	4.90	8.80±0.027	0.32
Interday	21.98±1.43	6.55	437,631.78±10,476.54	2.39	8.79±0.011	0.13

LOD, limit of detection; LOQ, limit of quantitation; RSD, relative standard deviation.

**Table 4.** Recovery of *p*-coumaroyltyramine (compound 10) from the sample

Amount spiked, μg/mL	Amount recovered (mean ± SD), μg/mL	% Recovery	% RSD
5	4.97±0.413	99.56±0.082	8.31
25	24.55±0.852	98.22±0.034	3.47
50	49.95±0.081	99.92±0.002	0.16

**Table 5.** The content of *p*-coumaroyltyramine (compound 10) in *C. sativa* roots of three different varieties determined by HPLC

Sample	Compound 10, μg/g	
	mean ± SD (*n* = 6)	% RSD
High CBD variety	19.78±0.728	3.68
Intermediate variety	8.00±0.348	4.35
High THC variety	7.65±0.359	4.70
